# Qualifying a Novel Clinical Trial Endpoint (iBOX) Predictive of Long-Term Kidney Transplant Outcomes

**DOI:** 10.3389/ti.2023.11951

**Published:** 2023-09-25

**Authors:** Amanda Klein, Alexandre Loupy, Mark Stegall, Ilkka Helanterä, Luke Kosinski, Eric Frey, Olivier Aubert, Gillian Divard, Kenneth Newell, Herwig-Ulf Meier-Kriesche, Roslyn Mannon, Thomas Dumortier, Varun Aggarwal, Jagdeep T. Podichetty, Inish O’Doherty, Ahmed Osama Gaber, William E. Fitzsimmons

**Affiliations:** ^1^ Critical Path Institute, Tucson, AZ, United States; ^2^ Institut National de la Santé et de la Recherche Médicale (INSERM), Paris, France; ^3^ Department of Surgery, Mayo Clinic, Rochester, Rochester, MN, United States; ^4^ Department of Transplantation and Liver Surgery, Helsinki University Central Hospital, Helsinki, Finland; ^5^ Division of Transplantation, Department of Surgery, Emory University School of Medicine, Atlanta, GA, United States; ^6^ Veloxis Pharmaceuticals, Cary, NC, United States; ^7^ Division of Nephrology, Department of Internal Medicine, College of Medicine, University of Nebraska Medical Center, Omaha, NE, United States; ^8^ Pharmacometrics, Novartis, Basel, Switzerland; ^9^ Department of Surgery, Houston Methodist Hospital, Houston, TX, United States

**Keywords:** kidney transplant, iBox, transplant outcomes, organ transplant, transplant clinical trial

## Abstract

New immunosuppressive therapies that improve long-term graft survival are needed in kidney transplant. Critical Path Institute’s Transplant Therapeutics Consortium received a qualification opinion for the iBOX Scoring System as a novel secondary efficacy endpoint for kidney transplant clinical trials through European Medicines Agency’s qualification of novel methodologies for drug development. This is the first qualified endpoint for any transplant indication and is now available for use in kidney transplant clinical trials. Although the current efficacy failure endpoint has typically shown the noninferiority of therapeutic regimens, the iBOX Scoring System can be used to demonstrate the superiority of a new immunosuppressive therapy compared to the standard of care from 6 months to 24 months posttransplant in pivotal or exploratory drug therapeutic studies.

## Graphical Abstract

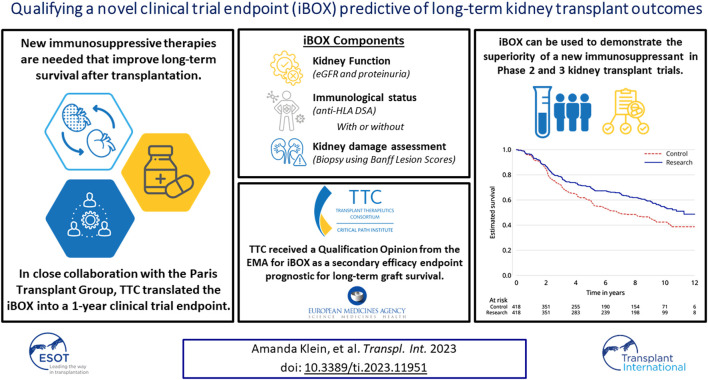

## Introduction

Graft failure following kidney transplantation has significant negative implications, including return to dialysis, lower life expectancy, decreased quality of life, and need for retransplantation. Additionally, graft survival is the most important outcome for people living with a kidney transplant [1]. Currently, immunosuppressive therapies (ISTs) have improved short-term outcomes in kidney transplantation, with 1 year graft survival rates of over 90% [2–5]. Despite the relatively low rate of efficacy failure at 1 year posttransplant, long-term graft survival remains suboptimal. The 5 and 10 years graft survival rates are 77% and 49% for deceased donor and 86% and 64% for living donor transplants [4]. Therefore, there remains a significant unmet need for ISTs that improve long-term outcomes. One of the challenges for biopharmaceutical sponsors is executing registration trials of a feasible size and duration (1–2 years) to support superiority claims using the historically accepted primary efficacy failure composite endpoint consisting of death, graft failure, biopsy-proven acute rejection, and lost to follow-up. These current endpoints, while acceptable to regulators, are not optimized for short-term superiority of ISTs that are predictive of longer-term graft survival. Such studies would require extended duration (e.g., 5 years or more), which may be impractical and unfeasible.

## Transplant Therapeutics Consortium (TTC)—A Regulatory-Focused Neutral Convener for Transplant

In 2014, the 2 major US transplantation societies, the American Society of Transplantation and the American Society of Transplant Surgeons, recognized the need for a pathway to develop new ISTs for transplant recipients [6]. In 2017, these societies partnered with Critical Path Institute and other transplant community members to create TTC (https://c-path.org/programs/ttc/). By facilitating a public–private partnership among scientists from the biopharmaceutical industry, diagnostics companies, academic institutions, professional societies, and government and regulatory agencies, TTC fosters consensus and data-driven research to increase speed in developing new therapies. TTC’s primary focus is obtaining regulatory endorsement of an early novel endpoint capable of predicting long-term graft survival in pivotal clinical trials designed to support regulatory approval of new ISTs for kidney transplantation.

**FIGURE 1:**
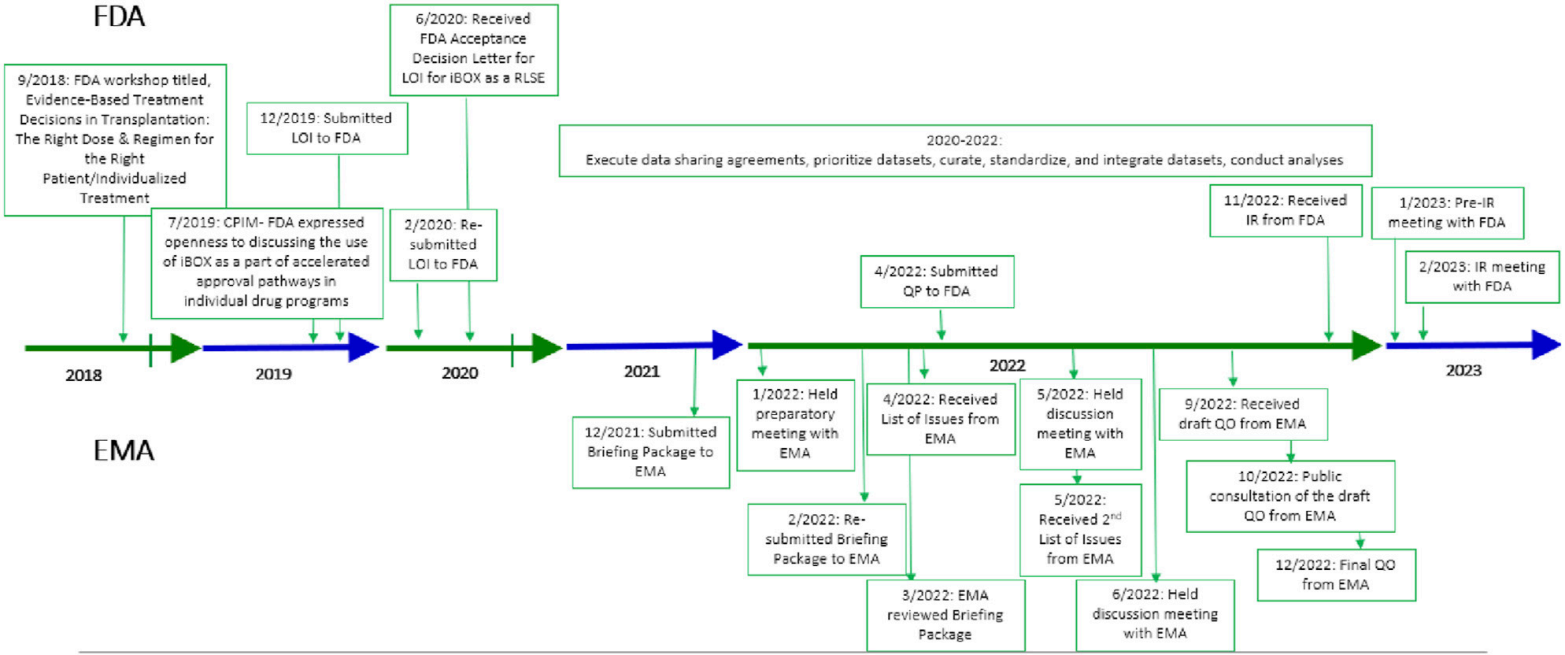
Regulatory timeline with FDA and EMA. CPIM, Critical Path Innovation Meeting; EMA, European Medicines Agency; FDA, Food and Drug Administration; IR, information request; LOI, letter of intent; QO, qualification opinion; QP, qualification plan; RLSE, reasonably likely surrogate endpoint.

To develop a novel trial endpoint, it is important to understand the multifactorial causes of late kidney graft failure; predicting failure accurately with a single marker may not be optimal [3]. Several composite scores have been proposed as surrogates, but iBOX is based on the largest dataset and the only specifically designed multivariate model that predicts long-term death-censored graft failure [7, 8]. iBOX is a risk prediction tool that utilizes multiple clinically relevant features demonstrated to be mechanistically associated with an increased risk of late graft functional decline and failure. These features are estimated glomerular filtration rate (eGFR), proteinuria, anti-human leukocyte antigen donor-specific antibody, and kidney graft biopsy histopathology measured cross-sectionally at any time point posttransplantation. iBOX then integrates these parameters to generate individualized predictions of outcomes at 3, 5, and 7 years posttransplant. iBOX was originally designed to be used at the patient level to inform clinical care and management of kidney transplant patients. In close collaboration with the Paris Transplant Group, TTC translated this work into a clinical trial endpoint acceptable to European Medicines Agency (EMA), intending to streamline drug development by predicting long-term outcomes using short-term data, summarized in Supplementary Table S1. Additionally, the qualification of iBOX as a reasonably likely surrogate endpoint (RLSE) is proceeding with the US Food and Drug Administration (FDA). The regulatory process and timeline associated with FDA and EMA interactions are shown in Figure 1.

## iBOX Scoring System–First Qualified Endpoint in Transplantation

In December of 2022, EMA issued a qualification opinion for iBOX as a secondary endpoint prognostic for death-censored graft loss in kidney transplant recipients intended to be used in clinical trials to support the evaluation of novel IST applications [9, 10]. EMA qualified both a full iBOX (including biopsy), and an abbreviated iBOX (excluding biopsy), allowing flexibility in using this endpoint in studies with and without protocol/surveillance biopsies. Importantly, the component measures in iBOX are modifiable by IST interventions and are further described in Table 1. The iBOX is the first qualified endpoint in transplantation and the fifth qualified endpoint with EMA [10].

**TABLE 1:** Component measures of the full and abbreviated iBOX.

iBOX component measures	Detailed information on the iBOX measures
Time of posttransplant risk assessment (fixed time points)	Phase 2/proof-of-concept iBOX assessment: 6 months
	Phase 3 iBOX assessment: 1 year, 2 years
Kidney function (eGFR and UPCR proteinuria)	eGFR, where eGFR is measured in mL/min/1.73 m^2^
	Log transformed (UPCR value^a^), where UPCR is measured in gram per gram (g/g)
Immunological status (anti-HLA DSA MFI)	Anti-HLA DSA using a qualitative binary MFI cutoff
	• MFI <1,400 (References group)
	• MFI ≥1,400
Kidney damage assessment^b^ (kidney allograft biopsy histopathology using Banff lesion scores)	Banff lesion score, interstitial fibrosis/tubular atrophy (IFTA score): Categorical variable with 3 levels
	• IFTA score = 0–1 (References group)
	• IFTA score = 2
	• IFTA score = 3
	Microcirculation inflammation (Banff lesion score, glomerulitis [g score] and Banff lesion score, peritubular capillaritis [ptc score]): Categorical variable with 3 levels
	• g and ptc score = 0–2 (References group)
	• g and ptc score = 3–4
	• g and ptc score = 5–6
	Banff lesion score, interstitial inflammation (i score) and Banff lesion score, tubulitis (t score): Categorical variable with 2 levels
	• i score and t score = 0–2 (References group)
	• i score and t score ≥3
	Banff lesion score, presence/extent of glomerular base membrane double contours; transplant glomerulopathy (cg score): Categorical variable with 2 levels
	• cg score = 0 (References group)
	• cg score = ≥1

DSA, donor-specific antibody; eGFR, estimated glomerular filtration rate; HLA, human leukocyte antigen; MFI, mean fluorescence intensity; UPCR, urine protein-to-creatinine ratio.

^a^ For proteinuria values below 0.05 g/g are replaced by 0.05 g/g before log-transformation.

^b^ Omitted from abbreviated iBOX.

An important outcome of this qualification is that iBOX can be used as a key secondary endpoint to demonstrate superiority of a new IST compared with the standard of care (SOC) from 6 months to 2 years posttransplant in exploratory or pivotal drug therapeutic studies for regulatory purposes. The datasets supporting this regulatory endorsement represent adult kidney-only transplant recipients with varying underlying diagnoses, multiple donor types, various induction therapies, and either calcineurin inhibitor (CNI)-based or CNI-free therapeutic regimens. As a result, iBOX can be used in registration-driven trials representative of a broad population of kidney transplant recipients. The context-of-use (COU) for iBOX is summarized in Table 2.

**TABLE 2:** Context-of-use for the qualification opinion of the iBOX Scoring System.

General measurement	The iBOX scoring system is a secondary endpoint prognostic for death-censored graft loss (allograft failure) in kidney transplant patients to be used in clinical trials investigating novel immunosuppressive medicines
Timing of iBOX assessments	The iBOX Scoring System is an acceptable secondary measured between 6 and 24 months postkidney transplantation in pivotal or exploratory drug therapeutic studies for regulatory purposes. The iBOX Scoring System can be used to demonstrate the superiority of a new immunosuppressive therapy compared with the SOC at 6, 12, or 24 months postkidney transplant
Target population	Adult kidney-only transplant recipients from a living or deceased donor

SOC, standard of care.

Additionally, in Europe, sponsors and investigators will be able to assess and promote the potential superiority of novel ISTs when measured using iBOX. Further, iBOX will be included in the summary of product characteristics, claims, and other product labeling. Although conditional marketing authorization (CMA) is a separate consideration outside the purview of the Qualification of Novel Methodologies for Drug Development process, superiority to current SOC, thereby addressing an unmet need in kidney transplant, is one of the key criteria for CMA in the European Union [8, 10].

## A Community-Based Approach to Endpoint Development

### Datasets

**FIGURE 2:**
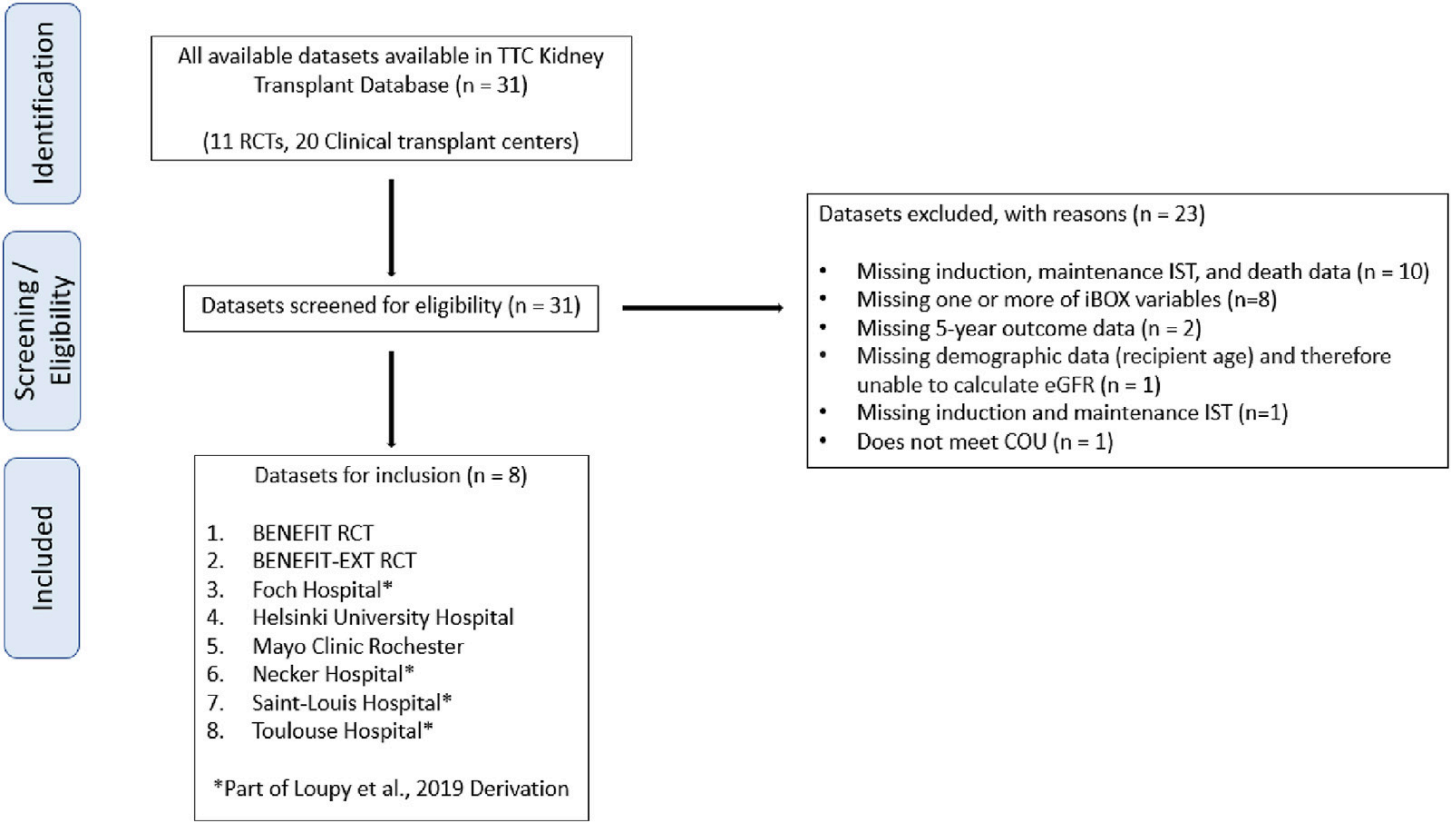
Flow diagram of the data selection process. COU, context-of-use; RCT, randomized controlled trial; TTC, Transplant Therapeutics Consortium.

A fundamental component of developing an evidentiary package that meets the requirements of regulatory endorsement for iBOX was the success of TTC’s extensive global patient-level data-sharing initiative [9–11]. Datasets from relevant clinical trials, including those used by [7] in their 2019 publication and real-world data from international clinical transplant centers, were prioritized for acquisition. A flow diagram of the dataset selection process is shown in Figure 2 with additional rationale provided in the Supplementary Material. The original iBOX [7] development included time posttransplant to account for varying iBOX assessments of individual patients and to assist in patient care and prognosis estimation. Figure 3 from [7] shows the density of iBOX risk evaluation time points after transplantation. The derivation dataset included in the EMA qualification submission represents all 4,000 subjects described in [7]. For application as a 1-year endpoint in a typical phase 3 clinical trial, we examined the number of subjects in the derivation dataset with iBOX assessments fixed at 1 year posttransplant and had outcome data of at least 5 years.

**FIGURE 3:**
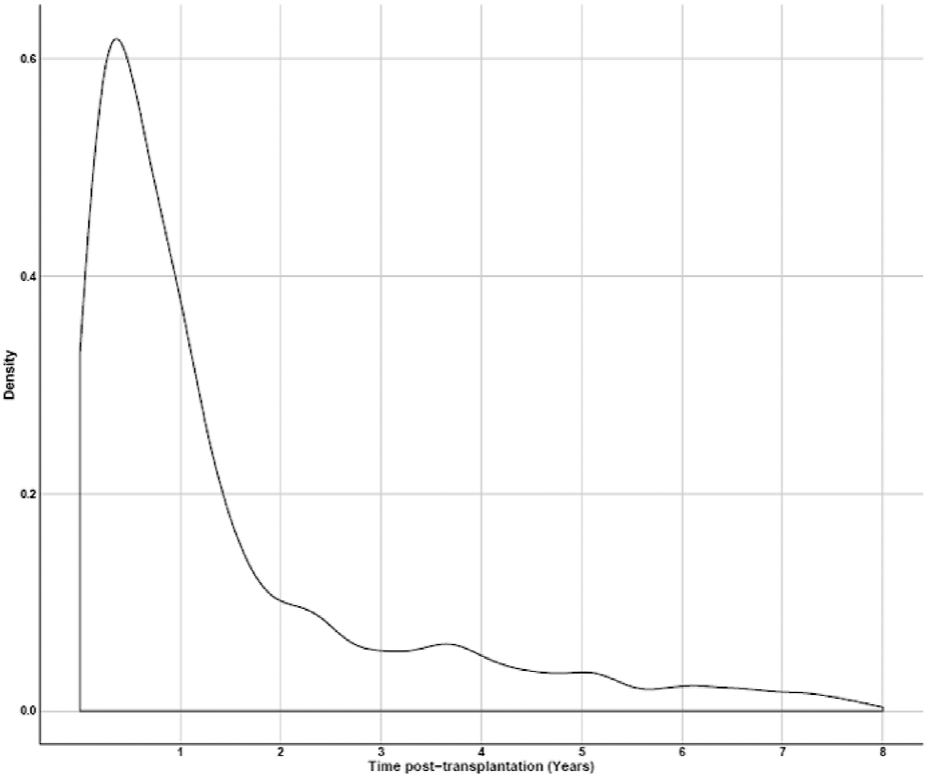
Density of time points where iBOX assessments were made (*y*-axis), compared with the time posttransplant (*x*-axis) out to 8 years, as shown in [7].

Five datasets supporting the regulatory endorsement of iBOX included data from clinical transplant centers (i.e., Loupy et al., 2019 derivation [7]. Mayo Clinic Rochester, and Helsinki University Hospital) and clinical trials (i.e., BENEFIT randomized controlled trial [RCT] [12] and BENEFIT-EXT RCT [13]) representing over 2,500 *de novo* kidney transplant recipients with 1-year iBOX assessments (Table 3). Participant consent was obtained from the transplant centers and clinical trials for primary uses. These datasets contained all elements necessary to assess the performance of iBOX as a pivotal trial endpoint including IST information, iBOX variables at 1 year posttransplant, and 5 years follow-up for death and graft loss of at least 5 years. Additionally, these datasets were accompanied by assay information for each component iBOX measure and laboratory certification documentation ensuring that the analytical methods were robust, reliable, and fit-for-purpose.

**TABLE 3:** Five-y posttransplant c-statistics values (SE) for the iBOX at 1 year posttransplant in the derivation and validation datasets.

Dataset		*n*	c-statistic (SE) for full iBOX at 1 year	c-statistic (SE) for abbreviated iBOX at 1 year
Derivation	[7] derivation	1174	0.85 (0.02)	NA
Validation	Mayo Clinic Rochester	483	0.93 (0.03)	0.84 (0.05)
	Helsinki University Hospital	344	0.78 (0.06)	0.77 (0.06)
	BENEFIT RCT	416	0.70 (0.09)	0.70 (0.08)
	BENEFIT-EXT RCT	260	0.81 (0.07)	0.78 (0.06)

NA, not applicable; RCT, randomized controlled trial; SE, standard error.

Clinical transplant center data are inherently heterogeneous and reflect the diversity of the kidney transplant recipient population in the United States and European Union. Datasets were curated, standardized, and aligned to conduct internal and external validation analyses to support the iBOX COU with EMA. Two clinical trial datasets have the most extensive CNI-free (belatacept [BELA]) patient-level data with the 4 core iBOX variables and sufficient follow-up period available. This represents, as stated by EMA, “extensive global effort to collect clinical trials and real-world data” [10].

Critical Path Institute explored the number of transplant recipients with full and abbreviated iBOX assessments at varying times posttransplant in the already curated and aligned validation datasets. Supplementary Figure S1 shows the distribution of assessment time points for donor-specific antibody (DSA) measurements up to 2 years postkidney transplant. DSA was selected for illustration because it is collected less frequently than eGFR and/or proteinuria and therefore acts as the key limiting factor for the availability of abbreviated iBOX measurements. The data distribution for iBOX assessments ranges from 6 months up to 2 years posttransplant. Helsinki University Hospital only assessed proteinuria and DSA data at 1 year posttransplant and therefore was excluded from the additional time points exploration. The number of transplant recipients with iBOX assessments at 6 months and 2 years posttransplant in the external validation datasets is shown in Supplementary Table S2. There were significantly more abbreviated iBOX assessments at the varying time points because biopsies were more typically “for-cause” and not taken “per protocol” at 6 months or 2 years posttransplant. Although the full iBOX measurements at 2 years were limited due to lack of biopsy information, because the abbreviated iBOX performed well at this time point, the addition of biopsy information should only further improve the performance, and therefore, the full iBOX is expected to also perform well at 2 years.

### Analyses

Validation analyses were performed to support the COU for iBOX with EMA for predicting death-censored graft loss. Both internal validations, evaluating iBOX on the data it was trained on (i.e., the derivation dataset), and external validation, evaluating iBOX on data it was not trained on, were performed. The abbreviated iBOX was treated as a modification of the full iBOX and not validated internally, save for checking the overall c-statistic. Both the full and abbreviated iBOX models were validated on 4 external datasets (i.e., validation datasets) (previously described above).

To avoid survivor bias, patients who did not reach their scheduled evaluation (i.e., those who lost their graft, died, or were lost to follow-up beforehand) were given an imputed worst-case iBOX score (Supplementary Tables S3, S4).

iBOX was validated by assessing its discrimination, the ability to rank individuals from a lower to a higher risk of graft loss, and its calibration, the ability to accurately predict absolute risk level [14]. Discrimination was assessed using Harrell’s c-statistic [15], which gives the probability that, for any 2 randomly selected individuals, the individual with the higher iBOX score, i.e., the higher model-predicted hazard of graft loss, has a shorter death-censored graft survival time. A c-statistic value of 0.7 or greater indicates good discriminatory ability [16]. Secondly, calibration was evaluated by checking whether observed events (graft losses) matched predicted using a Poisson calibration method (see Supplementary Material for a summary of the method) [14].

The full iBOX discrimination in the derivation dataset, when restricted to transplant recipients with an iBOX score at 1-year posttransplant and follow-up to 5 years, had a c-statistic of 0.85, demonstrating iBOX discriminates appropriately among subjects for use in a phase 3 study (Table 3). In the validation datasets, c-statistics ranged from 0.70 to 0.93 (Table 3), and the predicted versus observed graft losses were not significantly different for iBOX assessments at 1 year posttransplant (Table 4).

**TABLE 4:** Poisson calibration for the full and abbreviated iBOX at 1 year posttransplant in the validation datasets.

1 year Posttransplant				
Dataset	Full iBOX			
	*n*	Observed graft loss events	Predicted graft loss events	*p*
Mayo Clinic Rochester	483	18	24.34	.20
Helsinki University Hospital	344	21	14.40	.08
BENEFIT RCT	416	12	14.52	.51
BENEFIT-EXT RCT	260	12	14.97	.44

Dataset	Abbreviated iBOX			
	*n*	Observed graft loss events	Predicted graft loss events	*p*
Mayo Clinic Rochester	497	20	24.41	.37
Helsinki University Hospital	344	21	16.19	.23
BENEFIT RCT	515	15	18.77	.39
BENEFIT-EXT RCT	357	23	22.97	1.00

RCT, randomized controlled trial.

A *p*-value of <.05 would indicate a significant difference between the expected number of graft loss events as predicted by the iBOX versus the actual number of graft loss events.

Given that the iBOX models are trained primarily on subjects receiving CNI-based maintenance ISTs, it was unclear if iBOX would perform similarly in kidney transplant recipients not on CNI-based therapies. Internally, the iBOX was found to discriminate appropriately between higher- and lower-risk patients receiving mTOR inhibitor-based therapies (c-statistic >0.8) (Table 5). Externally, 5 years iBOX c-statistic values for CNI-free subjects, consisting primarily of patients on BELA-based regimens, at 1 year posttransplant in the validation datasets were evaluated; full and abbreviated iBOX c-statistics were 0.75 and 0.73, respectively (Table 6). These analyses demonstrate that iBOX can discriminate between subjects at higher and lower risk of death-censored graft loss in diverse datasets, including CNI and CNI-free populations, in clinical transplant centers and RCTs. Likewise, the results also showed that iBOX has good prediction accuracy based on calibration analyses (Table 6).

**TABLE 5:** Five-y posttransplant c-statistics values for the full iBOX for subset of subjects in the derivation dataset.

Subset of subjects in the [7] derivation	n	Observed graft loss events	c-statistic (SE)
mTORi subjects (includes subjects on both mTORi and CNI therapies)	239	33	0.87 (0.03)
mTORi-only subjects	171	23	0.86 (0.04)

CNI, calcineurin inhibitor; mTORi, mammalian target of rapamycin signal inhibitor; SE, standard error.

**TABLE 6:** Five-y posttransplant c-statistic values for the full and abbreviated iBOX for CNI and CNI-free subjects at 1 year posttransplant in the validation datasets.

Maintenance IST-based regimen	c-statistic (SE)	Observed graft loss events	Predicted graft loss events	*p*
Full iBOX				
CNI (TAC, CSA) *n* = 1045	0.82 (0.04) [TAC 0.86 (0.05), CSA 0.77 (0.05)]	50	51.6	.82
CNI-free (mTORi, BELA) *n* = 456	0.75 (0.08)^a^	13	16.6	.38
Abbreviated iBOX				
CNI (TAC, CSA) *n* = 1124	0.79 (0.04) [TAC 0.81 (0.05), CSA 0.77 (0.05)]	61	58.9	.78
CNI-free (mTORI, BELA) *n* = 587	0.73 (0.07)^a^	17	23.4	.26

BELA, belatacept; CNI, calcineurin inhibitor; CSA, cyclosporine; mTORi, mammalian target of rapamycin signal inhibitor; SE, standard error; TAC, tacrolimus.

^a^ The mTORi group only had 38 subjects with no graft loss events, so no breakdown of c-statistic by treatment was performed for the CNI-free group.

A *p*-value of <0.05 would indicate a significant difference between the expected number of graft loss events as predicted by the iBOX versus the actual number of graft loss events.

The performance of the full and abbreviated iBOX were also tested in the validation datasets at 6 months and 2 years posttransplant. The 5 years posttransplant discrimination (Supplementary Table S5) and calibration analyses (Supplementary Table S6) support the inclusion of time posttransplant in the iBOX model at 6 months and 2 years posttransplant.

Based on the iBOX formulas shown in Table 7, iBOX is not just the sum of the parts (i.e., the addition of components) but includes continuous and dichotomous variables weighted differently based on the beta coefficients. The c-statistic for eGFR alone and eGFR with proteinuria in comparison with the full and abbreviated iBOX is shown in Table 8, with calibration results in Supplementary Tables S7, S8, indicating that the iBOX score is influenced most by eGFR, and the other 3 components, proteinuria, anti-human leukocyte antigen DSA, and biopsy, all increase the predictive power.

**TABLE 7:** Formulas to calculate full and abbreviated iBOX scores.

	$iBox_{i}=\sum_{j=1}\hat{b_{j}}x_{i,j}$ for subject i where	Full iBOX	Abbreviated iBOX
	Factor	HR (exp $\left.{\left[\hat{\beta }\right.}_{j}]\right)$ (95% CI)^a^	
$X_{i,1}$	Time from transplant to evaluation (y)	1.08 (1.03–1.14)	1.12 (1.07–1.18)
$X_{i,2}$	eGFR (mL/min/1.73 m^2^)	0.96 (0.95–0.96)	0.95 (0.95–0.96)
$X_{i,3}$	Log transformed UPCR proteinuria (g/g)	1.5 (1.39–1.62)	1.59 (1.48–1.71)
$X_{i,4}$	Anti-HLA DSA MFI		
	<1,400	1	1
	≥1,400	1.84 (1.44–2.34)	1.84 (1.44–2.34)
$X_{i,5}$	Interstitial fibrosis/tubular atrophy (IFTA score)		N/A
	0–1	1	
	2	1.14 (0.92–1.43)	
	3	1.41 (1.1–1.8)
$X_{i,6}$	Microcirculation inflammation (g score and ptc score)	
	0–2	1
	3–4	1.43 (1.11–1.85)
	5–6	1.84 (1.25–2.7)
$X_{i,7}$	Interstitial inflammation and tubulitis (i score and t score)	
	0–2	1
	≥3	1.33 (1.06–1.68)
$X_{i,8}$	Transplant glomerulopathy (cg score)	
	0	1
	≥1	1.47 (1.14–1.9)

CI, confidence interval; DSA, donor-specific antibody; HLA, human leukocyte antigen; HR, hazard ratio; MFI, mean fluorescence intensity; N/A, not applicable.

^a^ ${\hat{\beta }}_{j}$ = the log of the HR values.

For categorical variables with more than 2 levels, e.g., IFTA score, the contribution of the variables was calculated as follows: β_1_x_1_ + β _2_x_2_. If the IFTA score = 0 or 1, then x_1_ = 0 and x_2_ = 0. If the IFTA score = 2, then x_1_ = 1 and x_2_ = 0. If the IFTA score = 3, then x_1_ = 0 and x_2_ = 1. β_1_ and β_2_ refer to the beta coefficients for the IFTA scores = 2 and 3, respectively.

**TABLE 8:** C-statistics for each validation dataset as parameters are removed in the iBOX with all parameters (“full”), without biopsy (“abbreviated”), without biopsy and DSA (“only eGFR and proteinuria”), and without biopsy, DSA, and proteinuria (“only eGFR”).

Dataset	c-statistic (SE) at 1 year posttransplant			
	Full iBOX	Abbreviated iBOX	iBOX with only eGFR and proteinuria	iBOX with only eGFR
Mayo Clinic Rochester	0.93 (0.03)	0.84 (0.03)	0.80 (0.04)	0.75 (0.04)
Helsinki University Hospital	0.78 (0.06)	0.77 (0.06)	0.76 (0.06)	0.74 (0.06)
BENEFIT RCT	0.70 (0.09)	0.70 (0.08)	**0.69 (0.08)**	**0.69 (0.08)**
BENEFIT-EXT RCT	0.81 (0.07)	0.78 (0.06)	0.78 (0.06)	0.78 (0.06)

eGFR, estimated glomerular filtration rate; DSA, donor-specific antibody; RCT, randomized controlled trial; SE, standard error.

Bold text highlights c-statistics <0.7.

In addition to validation, an analysis of the BENEFIT and BENEFIT-EXT RCTs included imputation of the worst-case iBOX scores at 1 year posttransplant for recipients who died or lost their graft in the first year (Table 9). This sensitivity analysis was performed to replicate the clinical trial setting where avoidance of survivor bias at 1 year would be necessary, and all randomized subjects would have an iBOX score at 1 year even if there were death or graft loss before that time. In both studies, the full and abbreviated iBOX score at 1 year was significantly lower in the BELA group than in cyclosporine, indicating a lower predicted risk of long-term graft failure. This corresponded to a statistically significant improvement in 5 years graft survival in the BENEFIT study. The BENEFIT-EXT study showed directionally higher 5 years death-censored graft survival. However, the difference was not statistically significant. The larger treatment difference in iBOX score at 1 year in the BENEFIT study compared with BENEFIT-EXT also corresponded to a larger treatment difference in graft survival. The lack of statistical significance on some of the 5 years graft survival analyses is related to limitations in the power to detect differences based on sample size.

**TABLE 9:** Treatment effect for 5 year graft survival with imputation (i.e., all-cause and death-censored) is the log HR, while the 1 year full and abbreviated iBOX scores are the difference in medians.

		BELA	CSA	Treatment effect	*p*
Full iBOX					
BENEFIT RCT (*n* = 466)	iBox score at 12 months: Median (SD)	−3.502 (0.07)	−2.915 (0.10)	−0.587	<.0001
	5 years KM survival probability % (SD)	96.0 (1.14)	89.7 (2.67)	−0.999	.02
BENEFIT-EXT RCT (*n* = 330)	iBox score at 12 months: Median (SD)	−2.6804 (0.065)	−2.1848 (0.12)	−0.4957	.0005
	5 years KM survival probability % (SD)	94.50 (1.55)	88.08 (3.43)	−0.8163	.071
Abbreviated iBOX					
BENEFIT RCT (*n* = 599)	iBOX score at 12 months: Median (SD)	−3.679 (0.05)	−3.042 (0.08)	−0.637	<.0001
	5 years KM survival probability % (SD)	96.3 (0.96)	89.7 (2.44)	−1.058	.006
BENEFIT-EXT RCT (*n* = 455)	iBOX score at 12 months: Median (SD)	−2.9057 (0.07)	−2.4255 (0.12)	−0.4803	.0007
	5 years KM survival probability % (SD)	85.05 (2.15)	78.54 (3.75)	−0.3292	0.2

BELA, belatacept; CSA, cyclosporine; KM, Kaplan-Meier; RCT, randomized controlled trial; SD, standard deviation.

Additional analyses were performed testing the performance of the full iBOX at 1 years posttransplant on all-cause 5 years graft loss (Supplementary Tables S9, S10). The discriminatory ability of iBOX for all-cause graft loss underperforms, with the full iBOX having reduced c-statistics, many of which are below 0.7, and poor all-cause calibration. This is expected given that iBOX was originally developed using variables more likely to impact risk of graft loss. Based on this evidence, iBOX was qualified with EMA with death-censored graft loss as the outcome measure.

## Sample Size Calculator Using iBOX Scores Using a Public-Facing Graphical User Interface

Separate from this EMA qualification submission, TTC developed a sample size calculator to assist sponsors in designing prospective clinical trials using iBOX as an endpoint. Sponsors can apply various inclusion/exclusion criteria and other specifications, consistent with the qualified COU, to calculate a sample size and project death-censored graft survival. This sample size calculator is publicly available at https://cpath.shinyapps.io/ibox_v3 to benefit the community and improve future clinical trial efficiency.

## Conclusion and Future Directions

The successful qualification opinion of iBOX by EMA is the first step in the process of providing an endpoint to allow the demonstration of superiority of new therapies and to stimulate the development of innovative therapies in kidney transplant. Validation analyses show that iBOX is suitable for predictions of graft loss events, with good performance based on c-statistics and the ability to predict numbers of graft loss events with reasonable margins of error, supporting the qualified COU with EMA. Although the original iBOX by [7] focused on the prognostic value for individual patient decision making, the tool was able to be adapted for regulatory purposes as a qualified clinical trial endpoint (Supplementary Table S1). iBOX as a secondary endpoint was put forward by EMA to further stimulate robust assessment of iBOX and may lend future opportunities to advance iBOX for other COUs, such as treatment of T cell-mediated or antibody-mediated rejection trials. Although this is an important step forward, it will not automatically lead to new innovative therapeutic development but must be applied strategically as an important tool in global development programs to demonstrate advantages over current SOC, which has good short-term results and is available as lower-cost generics.

Importantly, EMA has a higher evidentiary standard for qualifying a surrogate endpoint compared with the FDA. Unlike the FDA, EMA does not have a category of “reasonably likely” surrogate endpoints (RLSE), nor is CMA linked to surrogacy [17, 18] whereas the FDA has both an RLSE and an accelerated approval pathway that is based on surrogate endpoints. To facilitate the harmonization of multinational trials, TTC submitted the iBOX as an RLSE to the FDA Biomarker Qualification Program, and it is currently under review by the FDA [19]. Recent TTC interactions with the FDA have focused on the needs of transplant recipients for new innovative therapeutics that have demonstrated superiority to the current SOC and the inadequacy of relying solely or primarily on the historical efficacy failure endpoint, which is driven by acute rejection. Ideally, we envision designing one phase 3 *de novo* trial with iBOX as a primary endpoint in the United States for Accelerated Approval (i.e., RLSE) and a secondary endpoint in the European Union after establishing noninferiority for efficacy failure, alongside pursuing CMA. The ability to conduct trials with sites in the United States and the European Union is critical to advancing the field and bringing new and improved therapies to kidney transplant recipients. As stated by the EMA in the qualification opinion, “The Committee for Medicinal Products for Human Use encourages the use of the iBOX scoring system as a secondary endpoint in future trials of kidney transplantation and further development of the scoring system targeting a potential future qualification as a surrogate endpoint” [10].

## Figures and Tables

**Table:** 

*Critical Path Institute’s Transplant Therapeutics Consortium*	
Government and Regulatory Agencies	
• National Institutes of Health	
• U.S. Food and Drug Administration	
Academic Institutions	
• Charité – Universitätsmedizin Berlin	
• Helsinki University Hospital	
• Hospital do Rim	
• Houston Methodist Hospital	
• Katholieke Universiteit Leuven	
• Mayo Clinic	
• Necker Hospital, Paris Transplant Group	
• University of Colorado, Denver	
• University of Wisconsin, School of Medicine	
• University of Manitoba	
Professional Societies	
• American Society of Transplantation	
• American Society of Transplant Surgeons	
• The European Society for Organ Transplantation	
• The Transplantation Society	
Biopharmaceutical Industry and Diagnostic Companies	
• argenx	
• Bristol Myers Squibb	
• CareDx	
• CSL Behring	
• Eledon Pharmaceuticals	
• Eurofins Transplant Genomics	
• Hansa Biopharma	
• Immucor	
• Novartis	
• Sanofi	
• Takeda Pharmaceutical Company Limited	
• Talaris Therapeutics	
• Thermo Fisher Scientific Incorporated	
• Veloxis Pharmaceuticals	
*Additional Acknowledgments:*	
• Anat Tambur	• Klaus Romero
• Byron Smith	• Lauren Quinlan
• Cecile Olivier	• Mark Haas
• Christina Higgins	• Mike Pauley
• Christine Miller	• Nicholas King
• Devrim Eren	• Nicole Spear
• Erin Beach	• Patrick Lang
• Grace Erhart	• Rhoda Muse
• Hailey Davenport	• Sarah David
• Howie Gebel	• Stephen Karpen
• Jackson Burton	• Stephen Piccoli
• John Holman	• Zihan Cui
• Kitty Bogy	

## Data Availability

The aggregated dataset that was the basis for the work discussed in this publication is not publicly available as per requirements in the data contribution agreements. Requests to access these datasets should be directed to corresponding author.

## Abbreviations

BELA, belatacept; CMA, conditional marketing authorization; CNI, calcineurin inhibitor; COU, context-of-use; DSA, donor-specific antibody; eGFR, estimated glomerular filtration rate; EMA, European Medicines Agency; FDA, Food and Drug Administration; IST, immunosuppressive therapy; RCT, randomized controlled trial; RLSE, reasonably likely surrogate endpoint; SOC, standard of care; TTC, Transplant Therapeutics Consortium.
