# Supplementary material for: Qualifying a Novel Clinical Trial Endpoint (iBOX) Predictive of Long-Term Kidney Transplant Outcomes
Source: Transpl Int. 2023 Sep 25;36:11951. doi: 10.3389/ti.2023.11951 (PMC10563802; doi:10.3389/ti.2023.11951)
Supplement: Supplementary file 1 [file DataSheet1.pdf]

Supplemental document

A summary detailing the datasets in the TTC's Kidney Transplant Database with associated information describing why datasets were included/excluded in the EMA qualification submission is included below.

To acquire the subject-level data necessary to develop a novel surrogate endpoint, the TTC led an extensive global data collaboration effort across the field of kidney transplantation. To date, the TTC has acquired eleven clinical trial datasets and twenty observational datasets from clinical transplant centers, representing data from over 20,000 kidney transplant recipients in the TTC Kidney Transplant Database (Figure 2 in the main manuscript).

Datasets from relevant clinical trials of ISTs, including those in the Loupy et al. 2019 publication, and real-world data from international clinical transplant centers were prioritized for acquisition. From these 31 datasets, five contained all necessary variables collected at one-year post-transplant (i.e., eGFR, proteinuria, kidney allograft biopsy histopathology, and DSA), long-term death and graft loss follow-up of at least five years, immunosuppressive regimen information (i.e., induction and maintenance IST) to test the performance of the surrogate with all three MOA, and the documentation required to support the description of the analytical considerations for each dataset.

Datasets missing the necessary variables at one-year post-transplant or a variable necessary to calculate the model variable (as in recipient age to calculate an eGFR value) were excluded. For example, in the data for the three Novartis studies (TRANSFORM, US-92, and ELEVATE), recipient age was missing due to Novartis' anonymization procedures for data sharing. This, in turn, prohibited calculating eGFR values for the subjects in these studies. Moreover, US-92 and ELEVATE were missing DSA and proteinuria data, and follow-up was limited to one and two years, respectively.

Five datasets had the requisite subject-level data to conduct the internal and external validation analyses in this Briefing Dossier for a Qualification Opinion submission. These datasets were acquired from clinical transplant centers (i.e., Loupy et al., 2019 derivation, Mayo Clinic Rochester, and Helsinki

University Hospital) and clinical trials (i.e. [BENEFIT RCT] Vincenti et al., 2012 and [BENEFIT-EXT RCT] Medina-Pestana., 2012) representing over 5,500 *de novo* kidney transplant recipients. The subject-level data received from clinical transplant centers are inherently heterogeneous and reflect the diversity of the kidney transplant recipient population globally. In addition, the two clinical trials included in this qualification submission have the most extensive CNI-free patient-level data available with the four core variables and sufficient follow-up period.

Paris Transplant Group provided data from approximately 8,500 kidney transplant recipients from clinical transplant centers and clinical trial datasets spanning Europe, North America, and South America. However, not all of these datasets had the requisite variables to support this qualification submission, particularly treatment effects, as immunosuppressive regimen information (i.e., induction and maintenance IST) were missing (Figure 2 in the main manuscript). Included in this qualification submission as additional supporting data is the external validation previously performed by Loupy et al., 2019 using the three RCTs, CERTITEM, RITUX ERAH, and BORTEJECT, and the three European centers part of the European validation cohort. The raw patient-level data was not included in the qualification submission.

Supplemental Table 1. iBOX as described in Loupy et al., 2019 versus iBOX in Qualification Opinion.

	iBOX in Loupy et al., 2019	iBox in Qualification Opinion
**Core components of model**	1. $eGFR_{MDRD}$<br>2. Proteinuria: log transformed UPCR<br>3. Kidney allograft biopsy histopathology<br>4. DSA: Semiquantitative MFI associated with anti-HLA DSA<br>a. <500<br>b. ≥500-3000<br>c. ≥3000-6000<br>d. ≥6000<br>5. Time of post-transplant risk evaluation: at any time from transplant	1. $eGFR_{MDRD}$<br>2. Proteinuria: log transformed UPCR; imputation methodology included for datasets using other proteinuria measurements<br>3. Two iBox Scoring System models, one with and one without kidney allograft biopsy histopathology<br>4. DSA: Binary qualitative MFI associated with anti-HLA DSA[*]<br>a. <1400<br>b. ≥1400<br>5. Time of post-transplant risk evaluation: one-year post-transplant
**Application**	Individual decision-making	Surrogate endpoint in kidney transplantation clinical trials
**Derivation set**	Loupy et al., 2019	Loupy et al., 2019
**External validation sets**	Hôpital Hôtel Dieu, Nantes, France; Hospices Civils, Lyon, France; University Hospitals, Leuven, Belgium; Johns Hopkins Medical Institute, Baltimore, MD; the Mayo Clinic, Rochester, MN; and the Virginia Commonwealth University School of Medicine, Richmond, VA	Mayo Clinic Rochester[ǂ];<br>Helsinki University Hospital;<br>BENEFIT RCT;<br>BENEFIT-EXT RCT
**Methodology**	Semiparametric Cox PH model	Semiparametric Cox PH model
**Outcomes**	Death-censored allograft survival	Death-censored allograft survival
**Worst-case iBOX score imputation for use in de novo clinical trials (i.e., imputation for death, graft loss, LTFU in first year of transplant)**	No	Yes

ǂ Different dataset than in Loupy et al., 2019

* Changing the cut-off from four categories, as described in Loupy et al., 2019, to a binary presence or absence maintains consistency with the intended use of SAB assay as a qualitative test per the FDA approved 510(k) clearance.

Supplemental Table 2. Number of subjects with six months and two-year post-transplant iBOX assessments in the validation datasets.

Dataset	6 months full iBOX (n)	6 months abbreviated iBOX (n)	2-year full iBOX (n)	2-year abbreviated iBOX (n)
Mayo Clinic Rochester	NA	NA	NA	NA
Helsinki University Hospital*	NA	NA	NA	NA
BENEFIT RCT	30	527	12	476
BENEFIT-EXT RCT	31	383	5	328

* No longitudinal DSA or proteinuria data.

Supplemental Table 3. Worst-case iBOX score imputation.

**Time of post-transplant risk evaluation** (fixed time point)	For application in *de novo* phase 3 kidney transplant study:<br><br>**iBOX assessment fixed at one-year post-transplant**
**Kidney function** (eGFR and UPCR proteinuria)	eGFR, where eGFR is measured in ml/min/1.73m^2:<br><br>eGFR value set at 0 ml/min/1.73m^2
	Log transformed (UPCR value[1]), where UPCR is measured in g/g:<br><br>log UPCR value set at the maximum dipstick proteinuria-imputed score
**Immunological status** (anti-HLA DSA MFI)	DSA using a qualitative binary MFI cut-off:<br><br>DSA MFI set at maximum binary qualitative cut-off of ≥ 1,400
**Kidney damage assessment (omitted from abbreviated iBOX)** (kidney allograft biopsy histopathology using Banff lesion scores)	Interstitial fibrosis/tubular atrophy (IFTA score):<br><br>IFTA score set at maximum value of 3
	Microcirculation inflammation (g score and ptc score):<br><br>g score and ptc score set at maximum categorical value of > 4
	Interstitial inflammation and tubulitis (i score and t score):<br><br>i + t score set at a maximum categorical value of ≥ 3
	Transplant glomerulopathy (cg score):<br><br>cg score set at maximum categorical breakdown of ≥ 1

Supplemental Table 4. Imputed iBOX score calculation.

	**Imputed iBOX score calculation**
**Full iBOX**	0.0791+0.4069*log(3.236)+0.3432+0.6079+0.2886+0.3848+0.6080 = **2.79**
**Abbreviated iBOX**	0.1150+ 0.4652*log(3.236)+0.8164 = **1.48**

Supplemental Table 5. Five-year post-transplant c-statistics values for the full and abbreviated iBOX at six months and two years post-transplant in the validation datasets.

	**c-statistics (SE) at 6-months post-transplant**	**c-statistics (SE) at 2-years post-transplant**
	**Full iBOX**	
**Dataset**		
Mayo Clinic Rochester	NA	NA
Helsinki University Hospital*	NA	NA
BENEFIT RCT	0.84 (0.07)	NA
BENEFIT-EXT RCT	0.71 (0.11)	NA
	**Abbreviated iBOX**	
Mayo Clinic Rochester	NA	NA
Helsinki University Hospital*	NA	NA
BENEFIT RCT	**0.68 (0.08)**	0.73 (0.10)
BENEFIT-EXT RCT	0.72 (0.06)	0.76 (0.07)

*No longitudinal DSA or proteinuria data.

Bold text highlights c-statistics < 0.7.

Supplemental Table 6. Poisson calibration for the full and abbreviated iBOX at six months and two years post-transplant in the validation datasets.

Dataset	6-months post-transplant				2-years post-transplant			
	n	Observed graft loss events	Predicted graft loss events	p value	n	Observed graft loss events	Predicted graft loss events	p value
	**Full iBOX**							
Mayo Clinic Rochester	NA	NA	NA	NA	NA	NA	NA	NA
Helsinki University Hospital*	NA	NA	NA	NA	NA	NA	NA	NA
BENEFIT RCT	30	3	2.03	0.50	NA	NA	NA	NA
BENEFIT-EXT RCT	31	5	3.08	0.28	NA	NA	NA	NA
	**Abbreviated iBOX**							
Mayo Clinic Rochester	NA	NA	NA	NA	NA	NA	NA	NA
Helsinki University Hospital*	NA	NA	NA	NA	NA	NA	NA	NA
BENEFIT RCT	527	19	22.01	0.52	476	11	13.61	0.48
BENEFIT-EXT RCT	383	26	29.29	0.54	328	13	18.77	0.19

* No longitudinal DSA or proteinuria data.

A p-value <0.05 would indicate a significant difference between the expected number of graft loss events as predicted by the iBOX versus the actual number of graft loss events.

Supplemental Table 7. Calibration for iBOX with only eGFR and proteinuria.

Dataset	n	Observed graft loss events	Predicted graft loss events	Observed /Predicted	z score for observed /predicted	p value
Mayo Clinic Rochester	1114	45	57.93	0.78	-1.69	0.09
Helsinki University Hospital	346	22	17.22	1.28	1.15	0.25
BENEFIT RCT	518	15	20.39	0.74	-1.19	0.23
BENEFIT-EXT RCT	362	23	24.25	0.95	-0.25	0.80

A p-value <0.05 would indicate a significant difference between the expected number of graft loss events as predicted by the iBOX versus the actual number of graft loss events.

Supplemental Table 8. Calibration for iBOX with only eGFR.

**Dataset**	**n**	**Observed graft loss events**	**Predicted graft loss events**	**Observed /Predicted**	**z score for observed /predicted**	**p value**
Mayo Clinic Rochester	1214	51	76.50	0.67	-2.90	<0.01
Helsinki University Hospital	346	22	19.40	1.13	0.59	0.56
BENEFIT RCT	526	17	25.66	0.66	-1.70	0.09
BENEFIT-EXT RCT	383	23	29.59	0.78	-1.21	0.23

A p-value <0.05 would indicate a significant difference between the expected number of graft loss events as predicted by the iBOX versus the actual number of graft loss events.

Supplemental Table 9. Full iBOX c-statistics for death-censored and overall graft survival (including death with a functioning graft) in the validation datasets.

Dataset	c-statistic (SE) for full iBOX at 1-year using death-censored graft survival	c-statistic (SE) for full iBOX at 1-year using all-cause graft loss
Mayo Clinic Rochester	0.93 (0.03)	0.74 (0.05)
Helsinki University Hospital	0.78 (0.06)	**0.69 (0.04)**
BENEFIT RCT	0.70 (0.09)	**0.69 (0.06)**
BENEFIT-EXT RCT	0.81 (0.07)	**0.66 (0.05)**

Bold text highlights c-statistics < 0.7.

Supplemental Table 10. Full iBOX calibration for death-censored and overall graft survival in the validation datasets.

Dataset	Full iBOX at 1 year using death-censored graft survival				Full iBOX at 1 year using all-cause graft loss			
	**n**	**Observed graft loss events**	**Predicted graft loss events**	**p value**	**n**	**Observed graft loss events**	**Predicted graft loss events**	**p value**
Mayo Clinic Rochester	483	18	24.34	0.20	35	24.34	0.03	483
Helsinki University Hospital	344	21	14.40	0.08	46	14.40	<0.01	344
BENEFIT RCT	416	12	14.52	0.51	28	14.52	<0.01	416
BENEFIT-EXT RCT	260	12	14.97	0.44	41	14.97	<0.01	260

A p-value <0.05 would indicate a significant difference between the expected number of graft loss events as predicted by the iBOX versus the actual number of graft loss events.

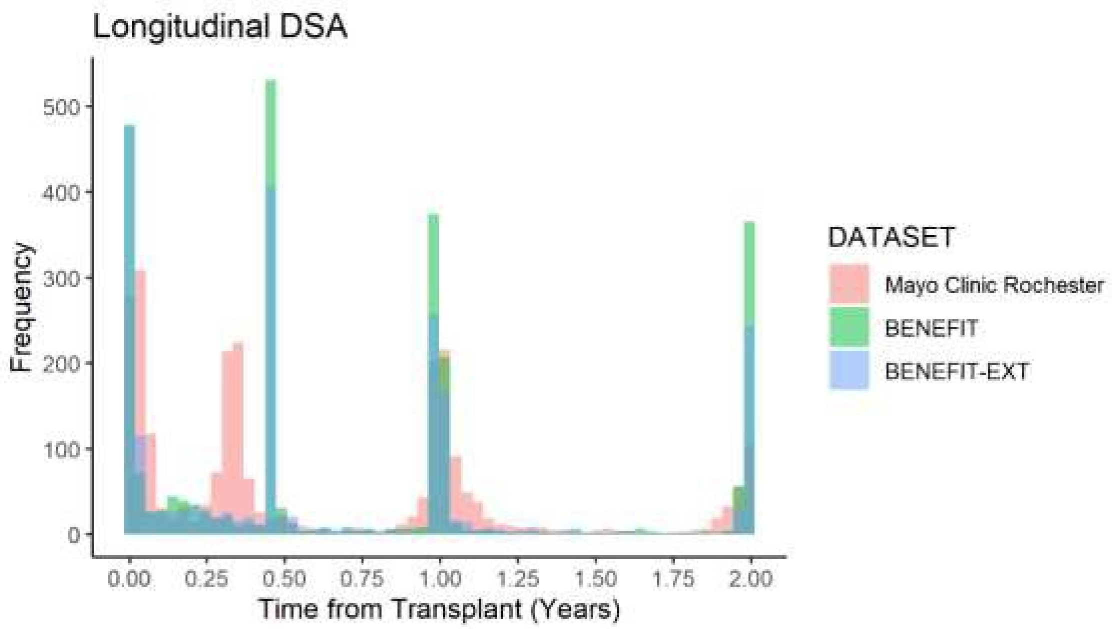

Supplemental Figure 1. Frequency of DSA measurements across the validation datasets.

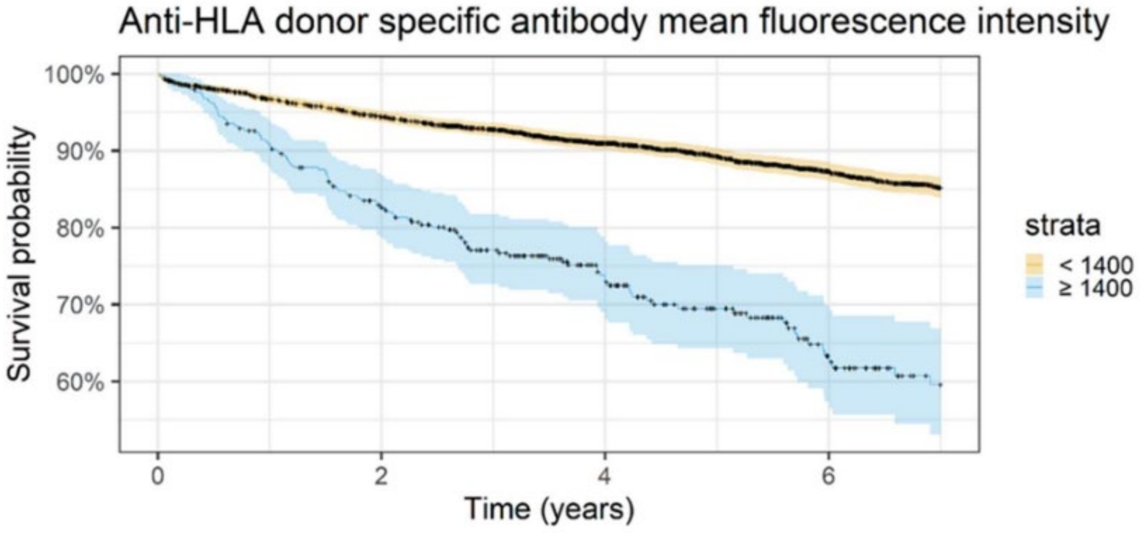

Supplemental Figure 2. Survival plot based on a binary anti-HLA DSA threshold of 1400 MFI.

Poisson calibration method

A cumulative hazard function $H(t)$, which can be calculated by integration from a hazard function $h(t)$, can be interpreted as the expected number of events experienced by time t. The calibration method described by Crowson et al. (2016) takes advantage of this property to assess the accuracy of the iBox Scoring System models for the external dataset using the following Poisson regression model:

$$\log(E[Y_i]) = \alpha + log\left(\widehat{H}_{iBox}(t_i, iBox_i)\right),$$

where Y_i is the number of events experienced by the ith subject of the dataset (in our case, 0 if the subject was censored and 1 if the subject experienced an event) during the observation period (from time 0 to t_i), $E[Y_i]$ is the expected number of events if this Poisson model is true, α is the model intercept, and $\widehat{H}_{iBox}(t_i, iBox_i)$ is the cumulative hazard at time of event or censoring t_i as predicted by the full and abbreviated iBox Scoring System for subject i as a function of its iBox score $iBox_i$. Here $log\left(\widehat{H}_{iBox}(t_i, iBox_i)\right)$ is used as an offset (a term where the coefficient is fixed to one) in the Poisson regression model.

The property mentioned above implies that $\alpha = 0$ if the iBox Scoring System model exactly predicts the number of events. Therefore, $\hat{\alpha}$ represents calibration-in-the-large, the degree to which the expected number of events predicted by the iBox Scoring System for the dataset subjects match the expected number of events predicted by the Poisson model (the latter of which is estimated using the actual number of observed events in the external dataset). Statistical significance is evaluated using the SE on this intercept term.

For additional methodological details, see Crowson et al. (2016).
